# MazEF Toxin-Antitoxin System-Mediated DNA Damage Stress Response in *Deinococcus radiodurans*

**DOI:** 10.3389/fgene.2021.632423

**Published:** 2021-02-19

**Authors:** Jingli Dai, Zijing Chen, Jinfeng Hou, Yudong Wang, Miao Guo, Jiajia Cao, Liangyan Wang, Hong Xu, Bing Tian, Ye Zhao

**Affiliations:** ^1^Institute of Biophysics, College of Life Sciences, Zhejiang University, Hangzhou, China; ^2^MOE Key Laboratory of Biosystems Homeostasis and Protection, Zhejiang University, Hangzhou, China

**Keywords:** toxin-antitoxin system, DNA damage, *Deinococcus*, ROS, iron transportation

## Abstract

*Deinococcus radiodurans* shows marked resistance to various types of DNA-damaging agents, including mitomycin C (MMC). A type II toxin-antitoxin (TA) system that responds to DNA damage stress was identified in *D. radiodurans*, comprising the toxin MazF-dr and the antitoxin MazE-dr. The cleavage specificity of MazF-dr, an endoribonuclease, was previously characterized. Here, we further investigated the regulatory role of the MazEF system in the response to DNA damage stress in *D. radiodurans*. The crystal structure of *D. radiodurans* MazF (MazF-dr) was determined at a resolution of 1.3 Å and is the first structure of the toxin of the TA system of *D. radiodurans*. MazF-dr forms a dimer mediated by the presence of interlocked loops. Transcriptional analysis revealed 650 downregulated genes in the wild-type (WT) strain, but not in the *mazEF* mutant strain, which are potentially regulated by MazEF-dr in response to MMC treatment. Some of these genes are involved in membrane trafficking and metal ion transportation. Subsequently, compared with the WT strain, the *mazEF* mutant strain exhibited much lower MMC-induced intracellular iron concentrations, reactive oxygen species (ROS), and protein carbonylation levels. These results provide evidence that MazEF-mediated cell death in *D. radiodurans* might be caused by an increase in ROS accumulation upon DNA damage stress.

## Introduction

Toxin-antitoxin (TA) modules, composed of a toxin gene and its cognate antitoxin, are widespread in most prokaryotic genomes (Ramisetty and Santhosh, 2017; Harms et al., 2018; Fraikin et al., 2020; Riffaud et al., 2020). Despite their implications is still a debatable topic, TA systems are linked to diverse biological processes, e.g., the stabilization of mobile elements through postsegregational killing, abrogation of bacteriophage infections through altruistic suicide, and formation of antibiotic-tolerant cells known as persisters (Nariya and Inouye, 2008; Van Melderen and De Bast, 2009; Slayden et al., 2018; Ronneau and Helaine, 2019). According to the nature and function of the toxins, TA systems can be divided into at least eight categories (Hayes and Van Melderen, 2011). Type I and type III antitoxins are RNA molecules that regulate the expression of toxin proteins by inhibiting either toxin mRNA translation or the toxin protein directly. While type II antitoxin proteins directly bind to and inhibit toxin proteins, type IV antitoxins indirectly counteract toxins, e.g., by reversing their effect on the targets.

In general, the MazEF system is an extensively studied type II TA module (Engelberg-Kulka et al., 2006; Ramisetty et al., 2015; Nikolic, 2019). The MazE antitoxin directly binds to the MazF toxin and forms a protein-protein complex, resulting in its neutralization. MazE is a short-lived protein *in vivo* and is degraded by proteases of the Clp family or by Lon under stress conditions. After MazE degradation, MazF is unleashed from the complex and acts as a sequence-specific endoribonuclease that cleaves RNAs, e.g., MazF in *Escherichia coli* recognizes the 3′ end of the first A base in an ACA sequence as the core cleavage site (Zhang et al., 2003; Munoz-Gomez et al., 2004). In addition, the MazF-mediated cell growth inhibition can be activated by various stress conditions, including antibiotics, high temperature, salt stress, DNA damage [caused by exposure to nalidixic acid, mitomycin C (MMC), or UV radiation], oxidative stress [caused by hydrogen peroxide (H_2_O_2_)] and amino acid starvation (Ramisetty et al., 2015; Lee and Lee, 2019). Moreover, it has been shown that the MazEF system plays an important role in biofilm formation in some bacteria (Kolodkin-Gal et al., 2015; Ma et al., 2019).

The crystal structures of MazF toxins from various bacteria have been determined. MazF proteins usually form homodimers, and the substrate RNA-binding site of the MazF protein is located near the dimeric interface, which undergoes a conformational change upon high-affinity binding of the MazE antitoxin protein (Kamada et al., 2003; Chen R. et al., 2020). Therefore, interactions between MazE and MazF exclude the RNA binding of MazF, leading to inhibition of the endoribonuclease activity of MazF protein.

*Deinococcus radiodurans* is an extremophilic bacterium that shows extraordinary resistance against environmental stressors, including ionizing radiation, ultraviolet radiation, oxidants, and MMC (Timmins and Moe, 2016; Lim et al., 2019; Chen Z. et al., 2020). Multiple mechanisms have been shown to contribute to the robustness of *D. radiodurans*, including strong cell defense systems, efficient DNA repair capability, and self-cleaning systems. In addition to the well-established DNA repair systems and antioxidation activities, a type II TA system, the MazEF-dr system, is found to promote dell death through the endoribonuclease activity of the MazF toxin in response to DNA damage in *D. radiodurans* (Li et al., 2017; Miyamoto et al., 2017). MazF-dr protein displayed cleavage preference for the RNA substrate, that is, at the nucleotide prior to the conserved ACA sequence with the order U > A > G > C. By searching the NCBI Reference Sequence Database, not all the *Deinococcus* species contain the MazEF system, indicating that presence of MazEF is not likely to be a byproduct of natural selection of environmental stressors but caused by the horizontal gene transfer events. Nevertheless, the detailed mechanism of MazEF-dr system-mediated DNA damage response in *D. radiodurans* remains unclear.

In the present study, the crystal structure of MazF-dr was determined. In addition to decreased MazE-dr protein levels *in vivo* under MMC stress, RNA-seq analyses of the wild-type (WT) and *mazEF* mutant strains of *D. radiodurans* upon MMC treatments revealed 650 downregulated genes that could potentially be regulated by the MazEF-dr system. Functional annotation analyses using the Gene Ontology (GO) and Kyoto Encyclopedia of Genes and Genomes (KEGG) databases suggested that these genes are enriched in membrane trafficking, metal ion transportation, and cell division. These data, together with biochemical studies, provide insights into MazEF-dr system regulation in response to DNA damage in *D. radiodurans*.

## Materials and Methods

### Growth Curve Measurements

Growth curves measurements were performed as previously described (Dai et al., 2020). WT and *mazEF* mutant strains were grown to early exponential phase, and then 100 μL cultures were resuspended in 50 mL fresh prepared TGY broth at 30°C. The optical density at 600 nm (OD_600_) was measured at every 4 h for a total incubation period of 40 h. Experiments were repeated three times.

### Quantitative Real-Time PCR (RT-qPCR)

Total RNA of both wild type and *mazEF* mutant strains were extracted as described (Dai et al., 2020) and then cDNA was amplified using SYBR Premix Ex Taq^TM^ GC (TaKaRa). RT-qPCR assays were performed using the Mx3005^TM^P Real-time detection system (Agilent, United States) and *groEL* was used to normalize RNA input. Experiments were repeated three times. RT-qPCR primers are listed in Supplementary Table S1.

### Strains, Plasmids and Growth Media

The *mazEF* (DR_0416-DR_0417) mutant strain was constructed as previously described (Supplementary Figures S1a,b) (Li et al., 2017). Compared with WT, cells devoid of *mazEF* gene showed similar cell growth under normal conditions (Supplementary Figure S1c). The transcriptional levels of two adjacent genes, DR_0415 and DR_0418, in both wild-type (WT) and *mazEF* mutant strains exhibited no significant difference under normal conditions (Supplementary Figure S1d), indicating that disruption of *mazEF* did not affect the functions of bordering genes. The *E. coli* strains used in this study were grown in lysogeny broth (LB) (1% tryptone, 0.5% yeast extract, and 1% sodium chloride) or LB agar at 37°C supplemented with 40 μg/mL kanamycin if necessary. All *D. radiodurans* strains were grown at 30°C in TGY broth (0.5% tryptone, 0.1% glucose, and 0.3% yeast extract) or on TGY agar, containing 30 μg/mL kanamycin or 10 μg/mL streptomycin if needed. The bacterial strains and plasmids used in this study are listed in Supplementary Table S1.

### Extraction of Total RNA From *D. radiodurans*

Wild-type and *mazEF* mutant cells were incubated to an OD_600_ of 0.8, centrifuged, and resuspended in PBS buffer (pH 7.0). Each suspension was divided in half: one half was treated with 15 μg/mL MMC for 30 min, and the other half was incubated without MMC under the same conditions. Total RNA of two biological replicates from each group was isolated using the TRIzol method according to the manufacturer’s protocol (Ambion, Foster City, CA, United States). Four micrograms of RNA per sample was used as the input material for library preparation, and then, a Ribo-Zero^TM^ Magnetic Kit (Epibio, MRZB12424) was used to remove rRNA. RNA concentration and RNA integrity were assessed using an RNA Nano 6000 Assay Kit and a Bioanalyzer 2100 system (Agilent Technologies, Santa Clara, CA, United States). Index codes were added to attribute sequences to each sample. The resultant libraries were sequenced on an Illumina HiSeq platform using the sequencing by synthesis (SBS) method and paired-end reads were generated. Raw reads were submitted to the NCBI Gene Expression Omnibus (GEO) under accession number GSE97563.

### RNA-Seq Data Analysis

The Kolmogorov–Smirnov test was used for the randomness test to ensure that the position distribution of the read length was distributed evenly. Differential expression analysis was performed using the differentially expressed genes (DEG) method (1.18.0), which provides statistical routines for determining differential expression in digital gene expression data using a negative binomial distribution model. Genes with adjusted *p*-values <0.05 were considered significantly differentially expressed.

Gene Ontology and KEGG pathway analyses were used to identify the enriched molecular functions and associated biological pathways of DEGs. The enrichment analyses were carried out through an online bioinformatics tool named The Database for Annotation, Visualization, and Integrated Discovery (DAVID2) (Huang et al., 2009). Only terms with a *p* < 0.05 were considered to be significant. Subcellular localization was analyzed using CELLO^1^ v.2.5.

### Protein Expression and Purification

MazF-dr was cloned into the expression vector pET28a+. *E. coli* BL21 (λDE3) pLysS cells transformed with expression vectors were cultured in LB with 40 μg/mL kanamycin. Protein expression and purification were performed according to a previously described method (Li et al., 2017).

### Crystallization, Data Collection, and Structure Determination

The MazF-dr protein containing an *N*-terminal 6× His-tag was concentrated to 5 mg/mL, and crystals were grown by the sitting-drop vapor diffusion method, using the Index^TM^-HR-144 kit (Hampton Research, Aliso Viejo, CA, United States) for initial screening at 293 K. MazF-dr crystals were optimized and obtained in the reservoir solution containing 0.2 M ammonium sulfate, 0.1 M Bis-Tris (pH 5.5), and 25% w/v polyethylene glycol 3350. Cryocooling was done by soaking the crystals in the reservoir solution containing 10, 20, and 30% glycerol for 3 min and flash freezing in liquid nitrogen. Diffraction intensities were recorded on beamline BL17U at the Shanghai Synchrotron Radiation Facility (Shanghai, China) and were integrated and scaled using the XDS suite (Kabsch, 2010). The structure was determined by molecular replacement using a published MazF structure (PDB ID: 6KYS) (Chen R. et al., 2020) as the search model. Structures were refined using PHENIX (Liebschner et al., 2019) and interspersed with manual model building using COOT (Emsley et al., 2010). Later stages of refinement utilized TLS group anisotropic B-factor refinement. MazF-dr forms a dimer in the crystallographic asymmetric unit. The refined structure includes 110 amino acids of MazF-dr (residues 4–113 from each protomer). All the residues were in the most favorable (97%) and allowed regions (3%) of the Ramachandran plot. All structural figures were rendered in PyMOL^2^.

### Western Blot Analysis

*Deinococcus radiodurans* strain was cultured in 500 mL of TGY broth at 30°C until the OD_600_ reached 0.8 and then divided into three parts, two of which were treated with 5 or 15 μg/mL MMC for 30 min, and the other part was untreated as a control group. Cells were then collected by centrifugation, washed twice with PBS, and resuspended in 3 mL of PBS for sonication. After centrifugation, the supernatant was subjected to SDS-PAGE using a 12% acrylamide resolving gel and electrotransferred onto a nitrocellulose membrane (0.22 μm, Pall Biotech, Shanghai, China). The membrane was blocked with 5% non-fat milk in 1× TBST and probed with anti-MazE-dr polyclonal antibody (homemade in the Animal Center of Zhejiang University of Traditional Chinese Medicine) overnight at 4°C, followed by HRP goat anti-rabbit antibody (1:10,000 dilution, Servicebio, Wuhan, China). The membrane was washed with TBST and developed using ECL reagents (Pierce, MA, United States) and imaged by exposure to light-sensitive film. The expression level of GroEL detected by a rabbit anti-GroEL polyclonal antibody was served as an internal control (Sigma, United States).

### Inductively Coupled Plasma Mass Spectrometry (ICP-MS)

Inductively coupled plasma mass spectrometry (ICP-MS) was performed as previously described (Dai et al., 2020). Cells were cultured in 500 mL of TGY broth until the OD_600_ reached 0.8, centrifuged and resuspended in TGY broth. Each suspension was divided in half: half was treated with 15 μg/mL MMC for 30 min, and the other half was incubated without MMC for the same duration. After harvesting, cells were washed using PBS containing 1 mM EDTA three times and then rinsed three times with PBS without EDTA. After drying for 12 h with a freeze-dryer (FD-1A-50, BIOCOOL, China), the cells were treated with 5 mL of Ultrex II nitric acid (Fluka AG., Buchs, Switzerland) and 1 mL of H_2_O_2_ at 100°C for 2 h. The concentrations of metal ions were measured by ICP-MS (ELAN DRC-e, PerkinElmer, United States).

### Measurements of Intracellular ROS Levels

Reactive oxygen species (ROS) levels were measured using 2′,7′-dichlorofluorescein diacetate (DCFH-DA) as a molecular probe. A 4-mL sample of cell cultures (OD_600_ = 0.8) was washed three times with PBS, resuspended in PBS containing 0.1 μm DCFH-DA, and incubated at 37°C for 30 min. After incubation, the cells were washed three times with PBS and resuspended in 4 mL of TGY. Each 1-mL sample was then treated with 10, 20, and 30 μg/mL MMC for 30 min, respectively, and a 1-mL sample without treatment was used as a control. For H_2_O_2_ treatment, each 1-mL sample was treated with 20, 40, and 80 mM H_2_O_2_ for 30 min, respectively, and a 1-mL sample without treatment was used as a control. DCFH-DA is hydrolyzed to DCFH by esterase and then oxidized by intracellular ROS to DCF, which produces fluorescence that can be measured using a fluorescence spectrophotometer (SpectraMax M5) at an excitation wavelength of 488 nm and an emission wavelength of 525 nm.

### Measurements of Protein Carbonylation Levels

Protein carbonylation was measured using the 2,4-dinitrophenyl hydrazine (DNPH) spectrophotometric method. Cell culture (OD_600_ = 0.8) was treated with 15 μg/mL MMC for 30 min, harvested, and resuspended in PBS. Cells were lysed using sonication, and the protein concentration in the cell-free extract was determined by the Bradford method. The cell extract was then incubated with 10 mM DNPH in 2 M HCl for 2 h in the dark. After precipitation by 10% trichloroacetic acid at 4°C, the precipitated proteins were washed three times with 50% ethyl acetate in ethanol. After evaporation, the protein sediments were dissolved in 6 M guanidine hydrochloride and centrifuged. The absorbance of the supernatant was determined at 370 nm against a protein control that had been processed in parallel but with 2 M HCl instead of DNPH. The protein carbonyl content was quantified in mmol/μg protein.

## Results

### Crystal Structure of MazF-dr

**FIGURE 1:**
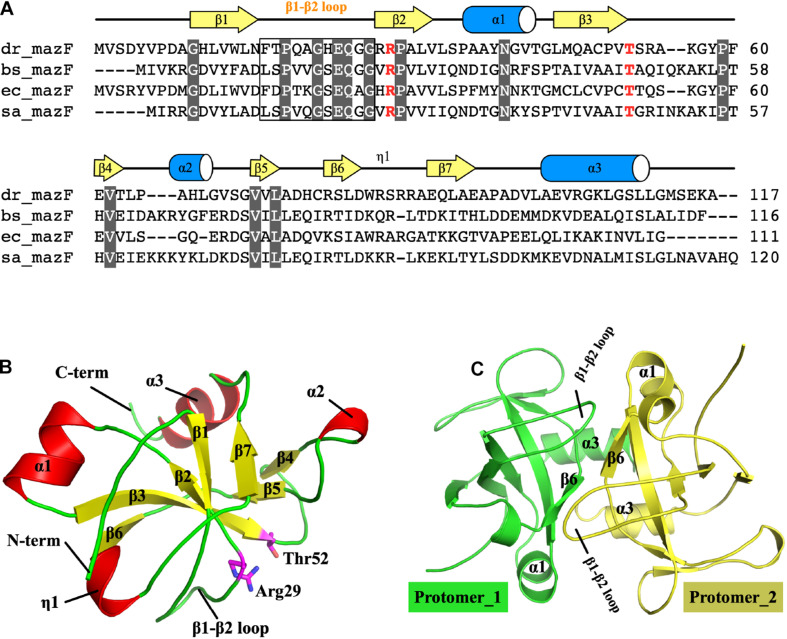
Structure of MazF-dr. **(A)** Structure-based sequence alignment of MazF proteins. MazF proteins from *D. radiodurans*, *B. subtilis*, *E. coli*, and *S. aureus* are denoted as dr_mazF, bs_mazF, ec_mazF, and sa_mazF, respectively. Residues conserved within MazF proteins are colored white. Two catalytic residues, namely, Arg29 and Thr52, are highlighted in red. **(B)** Structure of the MazF-dr protomer. Secondary structures, the β1–β2 loop and *N*- and *C*-termini are colored and labeled. Two catalytic residues, namely, Arg29 and Thr52, are shown as sticks and highlighted in magenta. **(C)** The MazF-dr dimer is shown as a cartoon, and the two protomers are colored green and yellow. Structural elements including the β1–β2 loop located at the dimer interface are labeled.

Sequence alignment revealed that MazF-dr has a similar overall domain arrangement as other bacterial MazF proteins, sharing 23% and 46% amino acid identity with the *Bacillus subtilis* and *E. coli* MazF proteins, respectively (Figure 1A). Crystal structure of full-length MazF-dr was determined at 1.3 Å resolution, which is the highest resolution currently known for MazF structures. MazF-dr crystallizes in space group *P*4_3_2_1_2 with a twofold symmetric dimer molecule in the crystallographic asymmetric unit. These two protomers could be well superimposed on each other with a root mean square deviation (rmsd) of 0.121 Å over 82 pairs of Cα atoms. The crystal data, together with the data collection and refinement statistics, are summarized in Table 1.

**TABLE 1:** Data collection, phasing, and refinement statistics.

	Native
**Data collection**	
Space group	*P*4_3_2_1_2
**Cell dimensions**	
*a*, *b*, *c* (Å)	70.49
	70.49
	111.33
Wavelength (Å)	1.000
Resolution (Å)	30.0–1.30
*R*_sym_ (%)	6.4 (58.1)
*I*/σ*I*	14.3 (2.7)
Completeness (%)	96.1 (99.0)
Redundancy	4.2 (4.2)
**Refinement**	
Resolution (Å)	30.0–1.30
No. reflections	69,092
*R*_work_/*R*_free_	17.2/18.5
**No. atoms**	
Protein	1,693
Ligand	15
Water	200
**B-factors**	
Protein	18.4
Ligand	47.0
Water	38.0
**R.m.s deviations**	
Bond lengths (Å)	0.018
Bond angles (°)	1.652

*Highest resolution shell is shown in parenthesis.*

Each protomer of MazF-dr is composed of three α-helices and seven β-strands, which form the typical MazF/CcdB fold frequently observed in published MazF structures (Figure 1B). As a typical MazF protein, MazF-dr could be aligned well with MazF homologs, including MazF from *E. coli* (PDB ID: 5CK9) (Zorzini et al., 2016), *B. subtilis* (PDB ID: 1NE8) (Gogos et al., 2003), and *Staphylococcus aureus* (PDB ID: 4MZM) (Zorzini et al., 2014) (Supplementary Figure S2). The rmsd of MazF-dr among these structures ranged from 0.469 to 1.347 Å over 58–73 Cα atoms, indicating the conserved overall conformation of MazF-dr. We performed the structure similarity search via DALI server using the entire MazF-dr structure as the query, and the highest score was for the MazF-mt1 protein from *Mycobacterium tuberculosis* (PDB ID: 6KYS, Supplementary Figure S2) (Chen R. et al., 2020). Similarly, the dimeric interface of MazF-dr calculated by the PISA server occupies a total buried surface area of 1,262 Å^2^ (21% of the total solvent-accessible surface area), suggesting the tight dimer formation of MazF-dr (Figure 1C). Two interlocked loops (β1–β2 loop) located at the dimeric interface between two protomers are fully ordered in the MazF-dr structure, in contrast to the high flexibility of this loop in its *E. coli* counterpart protein (Figure 1C and Supplementary Figure S2) (Kamada et al., 2003). Moreover, two catalytic residues, namely, Arg29 and Thr52 are well organized, suggesting the conserved ribonuclease activity of MazF-dr (Figure 1B).

### Transcriptional Analysis of the *D. radiodurans* Under MMC Treatments

The protein concentration of MazE *in vivo* is crucial to the regulation of the MazEF system under environmental stresses. Under normal conditions MazE could self-regulate the expression level of its operon, thus inhibiting the ribonuclease activity of MazF. Previously, we showed that the MazEF-dr system induced the cell death of *D. radiodurans* in response to a sublethal dose of MMC (Li et al., 2017). To confirm the degradation of MazE-dr under DNA damage, we checked the expression levels of MazE-dr *in vivo* under MMC treatments (Supplementary Figure S3). While strong immunoblot signals were detected under normal growth conditions, cells treated with 5 or 15 μg/mL MMC displayed decreased MazE-dr signals, implying the activation of the MazEF-dr system upon DNA damage.

**FIGURE 2:**
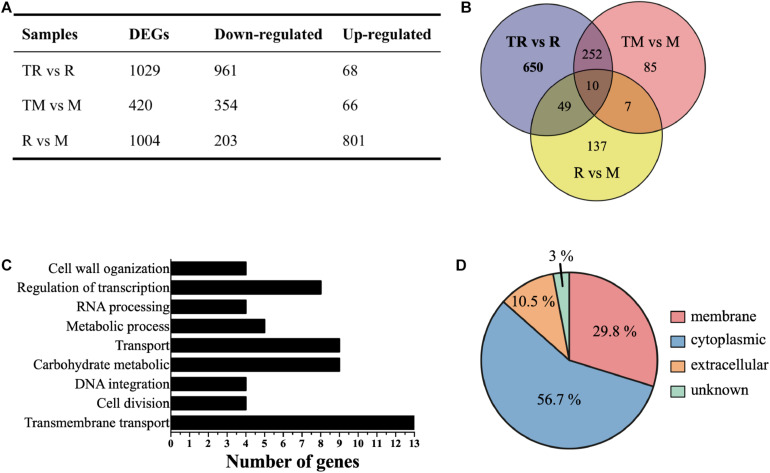
Transcriptional profiles of the wild-type (R) and *mazEF* mutant (M) strains under sublethal doses of MMC treatments. **(A)** Summary of differentially expressed genes (DEGs) in the wild-type treated with MMC vs the wild-type (TR vs R), the mutant treated with MMC vs the mutant (TM vs M) and the wild-type vs the mutant (R vs M). **(B)** The number of significantly downregulated genes potentially regulated by the MazEF-dr system plotted on a Venn diagram. **(C)** Histogram of biological process enrichment analysis of 650 selected downregulated genes. **(D)** Subcellular localization of 650 selected downregulated genes.

To further investigate the possible regulatory role of MazEF-dr-mediated DNA damage response, the transcriptomes of WT and *mazEF* mutant strains were analyzed in the presence or absence of MMC treatments using RNA sequencing. Four paired-end libraries were generated, yielding a total of 15.7, 15.7, 15.9, and 19.4 M clean reads from R (WT strain without MMC treatment), M (*mazEF* mutant strain without MMC treatment), TR (WT strain with MMC treatment), and TM (*mazEF* mutant strain with MMC treatment) with 150 bp as the average sequence length, which corresponded to 83.37–88.25% of the mapped genome of *D. radiodurans* (Supplementary Table S2). To explore the potential genes regulated by MazEF-dr upon MMC treatment, we compared the transcriptomes of TR vs R, TM vs M, and R vs M, respectively. Numbers of genes that were differentially expressed more than one-fold (log2) (*p* < 0.05) from each comparison group are summarized in Figure 2A. A total of 203 genes were downregulated and 801 genes were upregulated in the *mazEF* mutant strain compared with the WT strain under normal conditions (R vs M). In the presence of MMC treatment, a total of 1,029 DEGs were identified in the WT strain (TR vs R), including 961 downregulated genes and 68 upregulated genes. For the *mazEF* mutant strain (TM vs M), a total of 420 DEGs were identified under MMC treatment, with 354 downregulated genes and 66 upregulated genes. Given that potential genes regulated by the MazEF-dr system are expected to be cleaved by MazF-dr upon MMC treatment, we focused on the downregulated genes in TR vs R but not on those in R vs M or TM vs M to avoid overinterpretation of our data. For example, the transcriptional level of DR_A0320 (a putative urea transporter) was downregulated 2.25-fold (log2) in TR vs R but not significantly downregulated in TM vs M [0.13-fold (log2)] or R vs M [2.81-fold (log2)], suggesting that this gene was potentially regulated by the MazEF-dr system upon MMC treatment. As a result, a total of 650 downregulated genes in TR vs R were chosen as the potential genes regulated by the MazEF-dr system in response to MMC treatment in *D. radiodurans* (Figure 2B and Supplementary Data Sheet 1).

We next performed functional annotation analysis using the GO and KEGG databases. In the biological process category, these genes were involved in multiple biological processes including transmembrane transport, RNA processing, cell division, cell wall organization, regulation of transcription, and carbohydrate metabolism (Figure 2C). The results of KEGG pathway analysis revealed that these genes were mostly enriched in ATP-binding cassette (ABC) transporters participating in iron transportation, osmotic tolerance, and cell division (Table 2). These results suggested that the MazEF-dr system regulates gene expression upon MMC treatment in a variety of cellular processes. Moreover, approximately 30% of the proteins encoded by these genes were located in the cell membrane (Figure 2D), which may explain the observed markers of apoptotic cell death, e.g., membrane blebbing and loss of membrane integrity, in *D. radiodurans* upon MMC treatments (Li et al., 2017).

**TABLE 2:** Summary of genes enriched in ABC transporters. Genes belong to the same cluster were backgrounded with the same color.

Gene ID	Transport system	Subcellular localization	Description	TR vs R Log_2_ (fold)
DR_0462	Iron complex transport	Cytoplasmic	Substrate-binding protein	−1.38
DR_1550	Cell division transport	Membrane	Bacterial ftsEX	−1.05
DR_2192	Lipoprotein-releasing system	Cytoplasmic	ATPase activity	−1.34
DR_2469	Biotin transport	Cytoplasmic	ATPase activity	−1.17
DR_2470	Biotin transport	Membrane	Biotin binding	−1.36
DR_2488	Thiamine transport	Membrane	Permease protein	−1.02
DR_A0136	Osmoprotectant transport	Membrane	Permease protein	−2.15
DR_A0137	Osmoprotectant transport	Cytoplasmic	ATPase activity	−1.68
DR_A0320	Urea transport	Membrane	Substrate-binding protein	−2.25
DR_A0321	Urea transport	Membrane	Permease protein	−1.59
DR_A0324	Urea transport	Cytoplasmic	ATPase activity	−2.04
DR_A0349	Multidrug resistance transport	Membrane	Bacterial MsbA	−1.78
DR_B0014	Hemin transport	Membrane	Hemin-binding protein	−2.1
DR_B0016	Hemin transport	Cytoplasmic	ATPase activity	−1.54
DR_B0122	Iron complex transport (Fec)	Membrane	Bacterial FecD	−1.74
DR_B0123	Iron complex transport (Fec)	Membrane	Bacterial FecC	−2.57
DR_B0125	Iron complex transport (Fec)	Membrane	Bacterial FecB	−2.58

### The MazEF-dr System Regulates the Uptake of Fe Upon MMC Treatment

**FIGURE 3:**
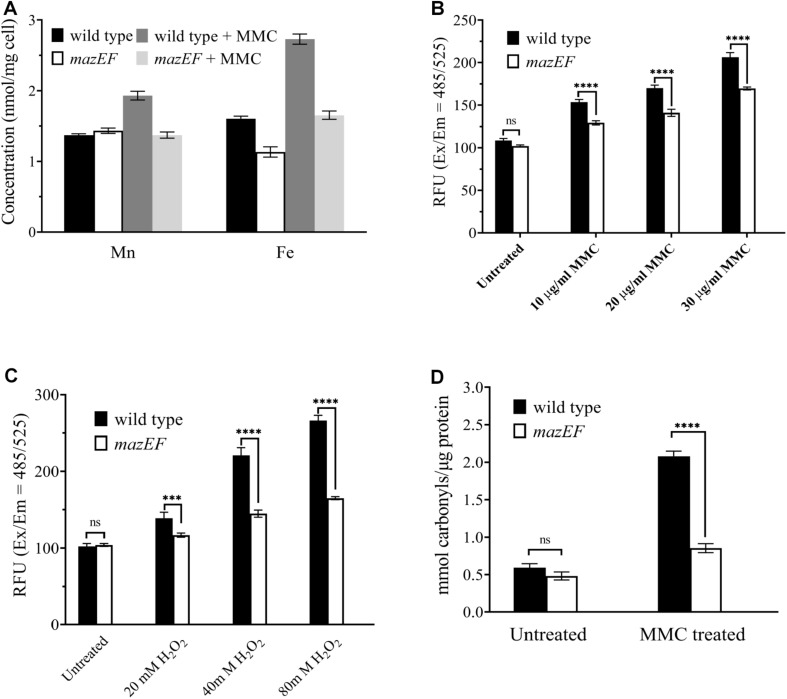
The MazEF-dr system induced an increased in intracellular Fe concentrations and ROS levels in response to MMC treatments. **(A)** ICP-MS analysis of the intracellular Mn and Fe concentrations in the WT and *mazEF* mutant cells with or without 15 μg/mL MMC treatments. **(B)** ROS levels of WT and *mazEF* mutant cells in the presence of 10, 20, and 30 μg/mL MMC. **(C)** ROS levels in WT and *mazEF* mutant cells in the presence of 20, 40, and 80 mM H_2_O_2_. **(D)** Protein carbonylation levels in WT and *mazEF* mutant cells measured by spectrophotometric DNPH assays. Cells were treated with 15 μg/mL MMC for 30 min. All the data represent the means of the three replicates (bars represent standard deviations. ns, not significant; ***p < 0.001, ****p < 0.0001).

Among all the 650 genes potentially regulated by the MazEF-dr system upon MMC treatment, we found that two sets of enriched genes in the KEGG pathway analysis, and these genes were involved in the iron complex transport system (Table 2). Three of these genes (DR_B0122, DR_B0123, and DR_B0125) encode the key components of the Fec system (FecBCD), which is important for ferrous iron transport. Two genes (DR_B0014 and DR_B0016) are involved in the transportation of the ferric-heme complex. Given that these genes were downregulated in the WT under MMC treatment, we next measured the intracellular concentrations of both Fe and Mn, using ICP-MS analyses (Figure 3A). Despite the similar intracellular concentration of Mn, the *mazEF* mutant strain had a 29.5% lower intracellular concentration of Fe than the WT strain in the absence of MMC. Upon MMC treatments, the intracellular concentrations of Fe and Mn in the WT strain (wild type + MMC in Figure 3A) increased by 70.1% and 40.9%, respectively. However, such elevation in metal ion concentrations was severely compromised in the *mazEF* mutant strain under MMC treatment (*mazEF* + MMC in Figure 3A), indicating that the MazEF-dr system plays a role in the increased intracellular concentrations of Mn and Fe upon MMC treatment.

### The MazEF-dr System Plays a Role in ROS-Mediated Cell Death

Excessive iron can cause damage to cellular components, e.g., proteins, through ROS formation via the Fenton reaction or Haber-Weiss reaction, which may further lead to cell death (Wardman and Candeias, 1996). Given the elevated intracellular Fe level in the WT strain upon MMC treatment, the total intracellular ROS levels were measured to further investigate the function of the MazEF-dr system in *D. radiodurans* (Figure 3B). The intracellular ROS levels in the WT strain increased with increasing concentrations of MMC, showing an MMC dose-dependent ROS accumulation profile (Figure 3B). In contrast, the ROS levels of the *mazEF* mutant strain were much lower than those of the WT strain, with compromised ROS accumulation upon MMC treatment. Similar results were observed when cells were treated with various concentrations of H_2_O_2_; much less ROS accumulation was observed in the *mazEF* mutant strain than in the WT strain (Figure 3C). These results were consistent with the distinct profile of Fe concentration in the WT and *mazEF* mutant strains in response to MMC treatments (Figure 3A). To further confirm the accumulation of ROS *in vivo*, protein carbonylation, the widely used marker of severe protein oxidation, in both the WT and *mazEF* mutant strains upon MMC treatment was measured (Figure 3D). Consistent with the increased ROS levels, high level of protein carbonylation (3.5-fold increase) was observed for the WT strain under MMC treatment. However, the *mazEF* mutant cells showed a significantly reduced level of protein carbonylation under MMC treatment. These results indicated that the MazEF-mediated cell death in *D. radiodurans* could be possibly caused by an increase in ROS accumulation upon DNA damage.

## Discussion

Toxin-antitoxin systems are widespread in many organisms and have attracted interest due to their multiple functions in response to environmental stressors, including DNA damage, heat shock and nutrient starvation. In addition to regulating cell growth, protein translation, and antibiotic resistance, MazEF system was reported to trigger PCD in *E. coli* MC4100 strain (Engelberg-Kulka et al., 2006; Lee and Lee, 2019). However, it should be noted that this PCD hypothesis is highly debated. A contradictory observation is that such MazEF-mediated PCD phenotype could not be detected in *E. coli* by several research groups (Tsilibaris et al., 2007; Ramisetty et al., 2016). *D. radiodurans*, containing a type II TA system, the MazEF-dr system, is well known for its extreme tolerance to DNA damage, including ionizing radiation and MMC (Lim et al., 2019). As a sequence-specific endoribonuclease recognizing the ACA motif, activation of MazF-dr led to the characteristics of apoptotic-like cell death upon sublethal doses of MMC treatments, while depletion of *mazEF* alleviated these phenotypes (Li et al., 2017).

In this study, we reported the high-resolution crystal structure of apo-MazF-dr. In the absence of substrate RNA or antitoxin MazE protein, MazF-dr forms a tight homodimer in the asymmetric unit, which adopts the canonical MazF/CcdB fold observed in published MazF structures (Figure 1). Structural superimposition of MazF-dr with other MazF structures revealed almost identical overall dimer conformations as well as active-site configurations, indicating the conserved catalytic mechanism of MazF-dr (Figure 1B and Supplementary Figure S2). To interact with the antitoxin MazE protein, the β1-β2 loop in *E. coli* MazF undergoes a disorder-to-order transition (Kamada et al., 2003). However, this loop is intact in the MazF-dr structure and interlocks the protein dimer, which was also observed in the *M. tuberculosis* MazF-mt1 structure, the most similar structure predicted by a DALI search (Supplementary Figure S2). Thus, it appears that a large conformational change or reorientation of this loop in MazF-dr toxin protein is required for the neutralization of MazF-dr by MazE-dr antitoxin.

Our data confirmed the decrease of the antitoxin MazE-dr upon MMC treatments, which could ultimately lead to the activation of the ribonuclease activity of MazF-dr toxin (Supplementary Figure S3). To further investigate the regulatory role of the MazEF-dr system, transcriptional profiles of the WT and *mazEF* mutant strains under sublethal doses of MMC treatments were compared, which revealed 650 downregulated genes (approximately 20.3% of the genome) potentially regulated by the MazEF-dr system in response to DNA damage stress in *D. radiodurans*. These genes are enriched in various biological processes, including cell growth and proliferation, RNA processing, protein translation, and cellular transportation. For example, genes involved in not only cell wall organization and cell division (DR_0297, DR_0627, DR_0844, DR_1090, and DR_0647) but also tRNA/rRNA processing (DR_2357 and DR_1694) were downregulated in the WT strain in the presence of sublethal doses of MMC. Moreover, the ABC transporter family FtsEX protein complex (DR_1550), which is widely conserved across diverse bacterial organisms, was downregulated (Meier et al., 2017; Alcorlo et al., 2020). FtsEX is involved in septal peptidoglycan synthesis and in the recruitment of cell division-related proteins (Du et al., 2020). Thus, the degradation of these gene transcripts by MazF-dr under MMC treatments inhibits cell proliferation, which could explain the cell growth arrest observed in MazF-dr-overexpression *E. coli* cells (Li et al., 2017).

In addition to the well-documented cleavage specificity of MazF, the cleavage of transcriptome by MazEF system depends on many factors, e.g., the genome sequence (abundance and locations of ACA motifs), transcriptional regulation by other factors, and various degree of stress conditions. And the transcriptional profile of MazF cleavage (as well as the function of MazEF) is possibly species-specific. In our case, despite that all the genes listed in Table 2 contain ACA sequences, not all of these 650 genes contain ACA site, e.g., DR_0092 and DR_0554. The expression of these genes under DNA damage were likely attenuated by indirect regulation of MazEF system. Thus, the MazEF-dr system may function as a global regulator under DNA damage stress in *D. radiodurans*, by combining direct (through RNA cleavage at ACA sites) and indirect (MazF-mediated gene down-regulation) mechanisms.

Notably, KEGG pathway analysis illustrated that these downregulated genes were enriched in ABC transporters, especially iron transporters, including genes involved in the Fec system, the non-heme pathway, and the ferric-heme transportation complex. The Fec system, the citrate-dependent iron transport system, was characterized as being involved in the cellular response to toxicity caused by excess intracellular ferrous ions (Staudenmaier et al., 1989; Braun and Braun, 2002). Ferric-heme transporters are related to the transportation of iron by some bacterial pathogens from mammalian hosts (Mazmanian et al., 2003). Given that intracellular concentration of iron depends on these heme- and non-heme-transportation systems, the MazEF-dr system is likely involved in the regulation of the intracellular Fe concentration in response to MMC treatments in *D. radiodurans*. It has been shown that the repression of heme enhanced the tolerance of *Porphyromonas gingivalis* persisters (Li et al., 2018), which may explain the apoptotic-like dell death of *D. radiodurans* cells in the presence of sublethal doses of MMC (Li et al., 2017).

To confirm the regulatory role of Fe ions in the MazEF-dr system, we measured intracellular Fe concentrations of the WT and *mazEF* mutant strains under sublethal doses of MMC treatments. In contrast to the *mazEF* mutant strain, the WT strains showed significant intracellular Fe ion accumulation in the presence of MMC, which might be due to the MazEF-dr system-mediated regulation of the Fe ion transportation. Indeed, in addition to these iron transporters, the Fec system and ferric-heme transport complex, the ferric uptake regulator gene (Fur, encoded by DR_0865) among the 650 gene candidates was also downregulated in the WT strain in response to MMC stress. In *D. radiodurans*, Fur serves as the repressor affecting both Mn and Fe uptake (Shah et al., 2014). Thus, in the presence of MMC stress, the downregulation of Fur transcripts mediated by the MazEF-dr system resulted in Fur derepression, allowing the uptake of excess metal ions, which is correlated with the highly increased intracellular concentrations of Mn and Fe in cells in response to MMC.

Accumulation of cellular Fe can lead to the overgeneration of ROS, which is toxic to living cells and might ultimately cause cell death. Not surprisingly, compared with the compromised ROS accumulation in the *mazEF* mutant strain, the WT strain had significantly higher ROS levels in response to both MMC and H_2_O_2_ treatments. Similar results were also observed for protein carbonylation levels under MMC stress conditions. In *E. coli*, two *mazEF*-mediated cell death pathways were proposed. While antibiotic-mediated inhibition of transcription or translation causes cell death in an ROS-dependent manner, DNA-damaging agents, e.g., nalidixic acid, induce ROS-independent cell death via the MazEF system. MMC was originally characterized as a natural antibiotic causing damage to DNA crosslinking. However, it can also interact with ribosomal RNA and inhibit protein translation (Snodgrass et al., 2010). Given the elevated ROS and protein carbonylation levels of the WT strain under MMC treatment, the activation of MazF-dr in response to MMC treatments appears to trigger ROS-mediated cell death in *D. radiodurans*. Notably, transcripts of key genes involved in DNA repair were not degraded by MazF-dr under MMC treatments (Supplementary Table S3), suggesting that the MazEF-dr system may not have effect on DNA repair processes in apoptotic-like cells.

In summary, we investigated the regulatory role of the MazEF-dr system in *D. radiodurans* in response to DNA damage. While the structure of MazF-dr is conserved among MazF proteins, the MazEF-dr system might induce ROS-dependent cell death by regulating various biological processes, including cell proliferation and Fe transportation.

## Data Availability Statement

All the data have been submitted to the NCBI Gene Expression Omnibus (GEO) under accession number GSE97563. The coordinates and structure factors have been deposited to Protein Data Bank with accession codes 7DHP.

## Author Contributions

YZ conceived the project. JD, ZC, and JH carried out crystallization and data collection. YZ and JD determined crystal structures and analyzed the data. JD, ZC, YW, and JC together performed the bioinformatic analyses and biochemical experiments. YZ, JD, and BT wrote the manuscript. All authors discussed the results, commented on the manuscript, and approved the final version to be published.

## Conflict of Interest

The authors declare that the research was conducted in the absence of any commercial or financial relationships that could be construed as a potential conflict of interest.

## Footnotes

1 http://cello.life.nctu.edu.tw/

2 www.pymol.org

## References

[B1] Alcorlo M. Straume D. Lutkenhaus J. Havarstein L. S. Hermoso J. A. (2020). Structural characterization of the essential cell division protein FtsE and its interaction with FtsX in *Streptococcus pneumoniae*. *Mbio* 11:e01488-20. 10.1128/mBio.01488-20 32873757 PMC7468199

[B2] Braun V. Braun M. (2002). Iron transport and signaling in *Escherichia coli*. *FEBS Lett.* 529 78–85. 10.1016/S0014-5793(02)03185-X 12354617

[B3] Chen R. Zhou J. Sun R. Du C. Xie W. (2020). Conserved conformational changes in the regulation of *Mycobacterium tuberculosis* MazEF-mt1. *ACS Infect Dis.* 6 1783–1795. 10.1021/acsinfecdis.0c00048 32485099

[B4] Chen Z. Tang Y. Hua Y. Zhao Y. (2020). Structural features and functional implications of proteins enabling the robustness of *Deinococcus radiodurans*. *Comput. Struct. Biotechnol. J.* 18 2810–2817. 10.1016/j.csbj.2020.09.036 33133422 PMC7575645

[B5] Dai J. L. Gao K. X. Yao T. Lu H. Z. Zhou C. L. Guo M. (2020). Late embryogenesis abundant group3 protein (DrLEA3) is involved in antioxidation in the extremophilic bacterium *Deinococcus radiodurans*. *Microbiol. Res.* 240:126559. 10.1016/j.micres.2020.126559 32721821

[B6] Du S. S. Pichoff S. Lutkenhaus J. (2020). Roles of ATP hydrolysis by FtsEX and interaction with FtsA in regulation of septal peptidoglycan synthesis and hydrolysis. *Mbio* 11:e01247-20. 10.1128/mBio.01247-20 32636250 PMC7343993

[B7] Emsley P. Lohkamp B. Scott W. G. Cowtan K. (2010). Features and development of coot. *Acta Crystallogr. D Biol. Crystallogr.* 66 (Pt. 4) 486–501. 10.1107/S0907444910007493 20383002 PMC2852313

[B8] Engelberg-Kulka H. Amitai S. Kolodkin-Gal I. Hazan R. (2006). Bacterial programmed cell death and multicellular behavior in bacteria. *PLoS Genet.* 2:e135. 10.1371/journal.pgen.0020135 17069462 PMC1626106

[B9] Fraikin N. Goormaghtigh F. Van Melderen L. (2020). Type II toxin-antitoxin systems: evolution and revolutions. *J. Bacteriol.* 202:e00763-19. 10.1128/jb.00763-19 31932311 PMC7167474

[B10] Gogos A. Mu H. Y. Bahna F. Gomez C. A. Shapiro L. (2003). Crystal structure of YdcE protein from *Bacillus subtilis*. *Proteins Struct. Funct. Bioinformatics* 53 320–322. 10.1002/prot.10457 14517982

[B11] Harms A. Brodersen D. E. Mitarai N. Gerdes K. (2018). Toxins, targets, and triggers: an overview of toxin-antitoxin biology. *Mol. Cell* 70 768–784. 10.1016/j.molcel.2018.01.003 29398446

[B12] Hayes F. Van Melderen L. (2011). Toxins-antitoxins: diversity, evolution and function. *Crit. Rev. Biochem. Mol. Biol.* 46 386–408. 10.3109/10409238.2011.600437 21819231

[B13] Huang D. W. Sherman B. T. Lempicki R. A. (2009). Systematic and integrative analysis of large gene lists using DAVID bioinformatics resources. *Nat. Protoc.* 4 44–57. 10.1038/nprot.2008.211 19131956

[B14] Kabsch W. (2010). Xds. *Acta Crystallogr. D Biol. Crystallogr.* 66(Pt 2) 125–132. 10.1107/S0907444909047337 20124692 PMC2815665

[B15] Kamada K. Hanaoka F. Burley S. K. (2003). Crystal structure of the MazE/MazF complex: molecular bases of antidote-toxin recognition. *Mol. Cell* 11 875–884. 10.1016/S1097-2765(03)00097-2 12718874

[B16] Kolodkin-Gal I. Verdiger R. Shlosberg-Fedida A. Engelberg-Kulka H. (2015). A differential effect of e. coli toxin-antitoxin systems on cell death in liquid media and biofilm formation. *PLoS One* 4:e6785. 10.1371/journal.pone.0006785 19707553 PMC2727947

[B17] Lee H. Lee D. G. (2019). Programmed cell death in bacterial community: mechanisms of action. Causes and Consequences. *J. Microbiol. Biotechnol.* 29 1014–1021. 10.4014/jmb.1904.04017 31216790

[B18] Li P. Fung Y. M. E. Yin X. H. Seneviratne C. J. Che C. M. Jin L. J. (2018). Controlled cellular redox, repressive hemin utilization and adaptive stress responses are crucial to metronidazole tolerance of *Porphyromonas gingivalis* persisters. *J. Clin. Periodontol.* 45 1211–1221. 10.1111/jcpe.13002 30125959

[B19] Li T. Weng Y. L. Ma X. Q. Tian B. Dai S. Jin Y. (2017). *Deinococcus radiodurans* toxin-antitoxin MazEF-dr mediates cell death in response to DNA damage stress. *Front. Microbiol.* 8:1427. 10.3389/fmicb.2017.01427 28798741 PMC5526972

[B20] Liebschner D. Afonine P. V. Baker M. L. Bunkoczi G. Chen V. B. Croll T. I. (2019). Macromolecular structure determination using X-rays, neutrons and electrons: recent developments in Phenix. *Acta Crystallogr. D Struct. Biol.* 75(Pt 10) 861–877. 10.1107/S2059798319011471 31588918 PMC6778852

[B21] Lim S. Jung J. H. Blanchard L. de Groot A. (2019). Conservation and diversity of radiation and oxidative stress resistance mechanisms in *Deinococcus* species. *FEMS Microbiol. Rev.* 43 19–52. 10.1093/femsre/fuy037 30339218 PMC6300522

[B22] Ma D. Z. Mandell J. B. Donegan N. P. Cheung A. L. Ma W. Y. Rothenberger S. (2019). The toxin-antitoxin MazEF drives *Staphylococcus aureus* biofilm formation, antibiotic tolerance, and chronic infection. *Mbio* 10:e01658-19. 10.1128/mBio.01658-19 31772059 PMC6879715

[B23] Mazmanian S. K. Skaar E. P. Gaspar A. H. Humayun M. Gornicki P. Jelenska J. (2003). Passage of heme-iron across the envelope of *Staphylococcus aureus*. *Science* 299 906–909. 10.1126/science.1081147 12574635

[B24] Meier E. L. Deitch A. K. Yao Q. Bhargava A. Jensen G. J. Goley E. D. (2017). FtsEX- mediated regulation of the final stages of cell division reveals morphogenetic plasticity in *Caulobacter crescentus*. *PLoS Genet.* 13:e1006999. 10.1371/journal.pgen.1006999 28886022 PMC5607218

[B25] Miyamoto T. Ota Y. Yokota A. Suyama T. Tsuneda S. Noda N. (2017). Characterization of a *Deinococcus radiodurans* MazF: a UACA-specific RNA endoribonuclease. *Microbiologyopen* 6:e501. 10.1002/mbo3.501 28675659 PMC5635168

[B26] Munoz-Gomez A. J. Santos-Sierra S. Berzal-Herranz A. Lemonnier M. Diaz-Orejas R. (2004). Insights into the specificity of RNA cleavage by the *Escherichia coli* MazF toxin. *FEBS Lett.* 567 316–320. 10.1016/j.febslet.2004.05.005 15178344

[B27] Nariya H. Inouye M. (2008). MazF, an mRNA interferase, mediates programmed cell death during multicellular *Myxococcus* development. *Cell* 132 55–66. 10.1016/j.cell.2007.11.044 18191220

[B28] Nikolic N. (2019). Autoregulation of bacterial gene expression: lessons from the MazEF toxin-antitoxin system. *Curr. Genet.* 65 133–138. 10.1007/s00294-018-0879-8 30132188 PMC6343021

[B29] Ramisetty B. C. Raj S. Ghosh D. (2016). *Escherichia coli* MazEF toxin-antitoxin system does not mediate programmed cell death. *J. Basic Microbiol.* 56 1398–1402. 10.1002/jobm.201600247 27259116

[B30] Ramisetty B. C. M. Natarajan B. Santhosh R. S. (2015). mazEF-mediated programmed cell death in bacteria: “What is this?”. *Crit. Rev. Microbiol.* 41 89–100. 10.3109/1040841x.2013.804030 23799870

[B31] Ramisetty B. C. M. Santhosh R. S. (2017). Endoribonuclease type II toxin-antitoxin systems: functional or selfish? *Microbiology (Reading)* 163 931–939. 10.1099/mic.0.000487 28691660

[B32] Riffaud C. Pinel-Marie M. L. Felden B. (2020). Cross-Regulations between bacterial toxin-antitoxin systems: evidence of an interconnected regulatory network? *Trends Microbiol.* 28 851–866. 10.1016/j.tim.2020.05.016 32540313

[B33] Ronneau S. Helaine S. (2019). Clarifying the link between toxin-antitoxin modules and bacterial persistence. *J. Mol. Biol.* 431 3462–3471. 10.1016/j.jmb.2019.03.019 30914294

[B34] Shah A. M. U. Zhao Y. Wang Y. F. Yan G. Q. Zhang Q. K. Wang L. Y. (2014). A mur regulator protein in the extremophilic bacterium *Deinococcus radiodurans*. *PLoS One* 9:e106341. 10.1371/journal.pone.0106341 25243898 PMC4171365

[B35] Slayden R. A. Dawson C. C. Cummings J. E. (2018). Toxin-antitoxin systems and regulatory mechanisms in Mycobacterium tuberculosis. *Pathog. Dis.* 76:fty039. 10.1093/femspd/fty039 29788125

[B36] Snodgrass R. G. Collier A. C. Coon A. E. Pritsos C. A. (2010). Mitomycin C Inhibits Ribosomal RNA A NOVEL CYTOTOXIC MECHANISM FOR BIOREDUCTIVE DRUGS. *J. Biol. Chem.* 285 19068–19075. 10.1074/jbc.M109.040477 20418373 PMC2885185

[B37] Staudenmaier H. Vanhove B. Yaraghi Z. Braun V. (1989). Nucleotide-Sequences of the Fecbcde genes and locations of the proteins suggest a periplasmic-binding-protein-dependent transport mechanism for Iron(Iii) dicitrate in *Escherichia*-Coli. *J. Bacteriol.* 171 2626–2633. 10.1128/jb.171.5.2626-2633.1989 2651410 PMC209944

[B38] Timmins J. Moe E. (2016). A decade of biochemical and structural studies of the DNA repair machinery of *Deinococcus radiodurans*: major findings, functional and mechanistic insight and challenges. *Comput. Struct. Biotechnol. J.* 14 168–176. 10.1016/j.csbj.2016.04.001 27924191 PMC5128194

[B39] Tsilibaris V. Maenhaut-Michel G. Mine N. Van Melderen L. (2007). What is the benefit to *Escherichia coli* of having multiple toxin-antitoxin systems in its genome? *J. Bacteriol.* 189 6101–6108. 10.1128/jb.00527-07 17513477 PMC1951899

[B40] Van Melderen L. De Bast M. S. (2009). Bacterial toxin-antitoxin systems: more than selfish entities? *PLoS Genet.* 5:e1000437. 10.1371/journal.pgen.1000437 19325885 PMC2654758

[B41] Wardman P. Candeias L. P. (1996). Fenton chemistry: an introduction. *Radiat. Res.* 145 523–531. 10.2307/3579270 8619017

[B42] Zhang Y. L. Zhang J. J. Hoeflich K. P. Ikura M. Qing G. L. Inouye M. (2003). MazF cleaves cellular mRNAs specifically at ACA to block protein synthesis in *Escherichia coli*. *Mol. Cell* 12 913–923. 10.1016/S1097-2765(03)00402-7 14580342

[B43] Zorzini V. Buts L. Sleutel M. Garcia-Pino A. Talavera A. Haesaerts S. (2014). Structural and biophysical characterization of *Staphylococcus aureus* SaMazF shows conservation of functional dynamics. *Nucleic Acids Res.* 42 6709–6725. 10.1093/nar/gku266 24748664 PMC4041440

[B44] Zorzini V. Mernik A. Lah J. Sterckx Y. G. J. De Jonge N. Garcia-Pino A. (2016). Substrate recognition and activity regulation of the *Escherichia coli* mRNA endonuclease MazF. *J. Biol. Chem.* 291 10950–10960. 10.1074/jbc.M116.715912 27026704 PMC4900246

