# Supplementary material for: MazEF Toxin-Antitoxin System-Mediated DNA Damage Stress Response in Deinococcus radiodurans
Source: Front Genet. 2021 Feb 19;12:632423. doi: 10.3389/fgene.2021.632423 (PMC7933679; doi:10.3389/fgene.2021.632423)

**MazEF toxin-antitoxin system-mediated DNA damage stress response in *Deinococcus radiodurans***

Jingli Dai^1,2,3^, Zijing Chen^1,2,3^, Jinfeng Hou^1,2^, Yudong Wang^1,2^, Miao Guo^1,2^, Jiajia Cao^1,2^, Liangyan Wang^1,2^, Hong Xu^1,2^, Bing Tian^1,2^, & Ye Zhao^1,2 *^

^1.^ Institute of Biophysics, College of Life Sciences, Zhejiang University, Hangzhou, Zhejiang, China

^2.^ MOE Key Laboratory of Biosystems Homeostasis & Protection, Zhejiang University, Hangzhou, Zhejiang, China

^3.^ These authors contributed equally to this work

Running title: MazEF-mediated stress response

* To whom correspondence should be addressed. Tel: 86-571-86971279; fax: 86-571-86971703; email: Z. Y. (yezhao@zju.edu.cn)

**Table S1. List of strains, plasmids, and primers used in this study.**

|  | Description | Resource |
| --- | --- | --- |
| Strains |  |  |
| *E. coli* |  |  |
| BL-21(λDE3), pLysS | *F^-^ ompT hsdSB (rB - mB -) gal dcm*  *λ(DE3) pLysS Camr* | TransGen Biotech |
| DH5ɑ | *F-φ80 lac ZΔM15 Δ (lacZYA-arg F) U169 endA1 recA1 hsdR17 (rk^-^, mk^+^) supE44λ-thi-1 gyrA96 relA1 phoA* | TransGen Biotech |
| *Deinococcus radiodurans* |  |  |
| R1 | wild type strain; ATCC:13939 | Laboratory Collection |
| *mazEF* | *mazEF* mutant strain | Laboratory Collection |
| Plasmids |  |  |
| pET28a+ | Expression vector containing 6×His-tag | Novagen |
|  |  |  |
| Primers for RT-qPCR | Sequence (5ʹ to 3ʹ) |  |
| groEL_F | AAGGGGCCTCCAAGCACAT |  |
| groEL_R | GGTCGAGGTGGGAATGA |  |
| DR_0415_F | CAGGGCCAGAGCCTCAAGGT |  |
| DR_0415_R | GGTCGGGCAGGCAAACACT |  |
| DR_0418_F | GTGAGTGACGCGGAGGGC |  |
| DR_0418_R | TCTCGCAGGCGGCGGTAG |  |

**Table S2 Summary of sequence reads statistics obtained from Illumina deep.** R, wild type strain without MMC treatment; M, *mazEF* mutant strain without MMC treatment; TR, wild type strain with 15 μg/mL MMC treatment; TM, *mazEF* mutant strain with 15 μg/mL MMC treatment.

| **Mapper RNAs Type** | **R** | **M** | **TR** | **TM** |
| --- | --- | --- | --- | --- |
| Clean reads | 15793356 | 15734556 | 15933956 | 19407451 |
| Matched genome | 13167626 | 13416489 | 14058479 | 16811619 |
| Matched genome (%) | 83.37% | 85.26% | 88.25% | 86.56% |
| protein-coding genes(sense) | 87.53% | 89.92% | 87.26% | 86.94% |
| protein-coding genes(antisense) | 4.42% | 2.16% | 2.75% | 2.21% |
| unannotated | 7.58% | 7.47% | 9.96% | 10.86% |
| ORFs | 3165 | 3150 | 3151 | 3151 |

**Table S3 Transcripts of important genes involved in DNA repair pathways, including Homologous recombination (HR), Single-strand Annealing (SSA), and nucleotide excision repair (NER).**

| **Gene** | **Description** | **Pathway or function** | **TR vs R Log2(fold)** | **TM vs M Log2(fold)** |
| --- | --- | --- | --- | --- |
| DR_2340 | RecA | HR | 0.30 | 0.86 |
| DR_1126 | RecJ | HR | 0.30 | 0.02 |
| DR_0070 | ddrB | SSA | 0.28 | 0.31 |
| DR_0423 | ddrA | DNA end-protection | -0.18 | -0.54 |
| DR_1089 | RecF | HR | 0.24 | 0.03 |
| DR_0198 | RecR | HR | 0.14 | 0.24 |
| DR_1289 | RecQ | HR | -0.67 | -0.63 |
| DR_0819 | RecO | HR | -0.26 | 0.19 |
| DR_1477 | RecN | HR | -0.75 | -0.41 |
| DR_1775 | UvrD | HR | -0.47 | -0.36 |
| DR_A0346 | PprA | Promotes DNA repair | 0.17 | -0.28 |
| DR_1771 | UvrA1 | NER | 0.62 | 0.45 |
| DR_2275 | UvrB | NER | 0.63 | 0.48 |
| DR_1354 | UvrC | NER | 0.34 | -0.08 |
| DR_1913 | GyrA | Topoisomerases | 0.25 | 0.41 |
| DR_0906 | GyrB | Topoisomerases | 0.75 | 0.83 |

**Figure S1.** Construction of *mazEF* knock-out mutant. (a) Schematic representation of the gene mutation by homologous recombination which replaced the DR_0416-DR_0417 operons with streptomycin-resistant cassette. (b) DNA sequence of *mazEF* mutant strain. DR_0415, streptomycin-resistant cassette, and DR_0418 are highlighted in distinct colors. BamH1 and HindIII restriction site are highlighted in cyan and purple, respectively. (c) Growth curves of WT and *mazEF* mutant strains. Cells were individually grown in TGY medium, and growth rates were recorded by measuring the OD_600_ every 4 h. (d) Quantitative real-time PCR analysis of the gene expression levels of DR_0415 and DR_0418 in the WT and *mazEF* mutant strains under normal growth conditions. The data represent the means of the three replicates, and the bars represent their standard deviations (ns: not significant).

**
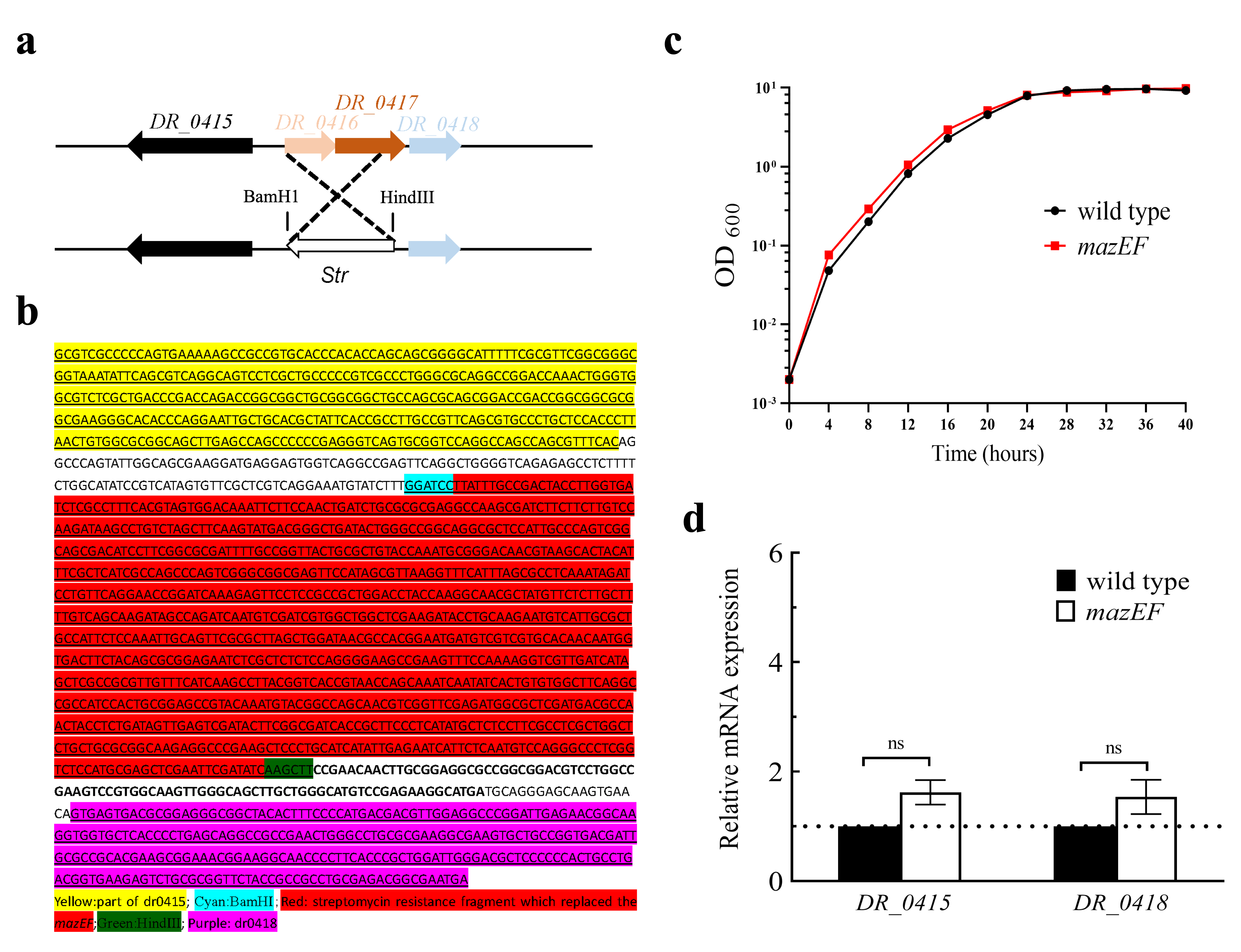
**

**Figure S2.** Superimpositions of the MazF structures from *D. radiodurans* (yellow), *B. subtilis* (PDB ID: 1NE8, blue white), *E. coli* (PDB ID: 5CK9, green), *M. tuberculosis* (PDB ID: 6KYS, cyan), and *S. aureus* (PDB ID: 4MZM, pink). β1-β2 loop showing noticeable deviations are labelled.


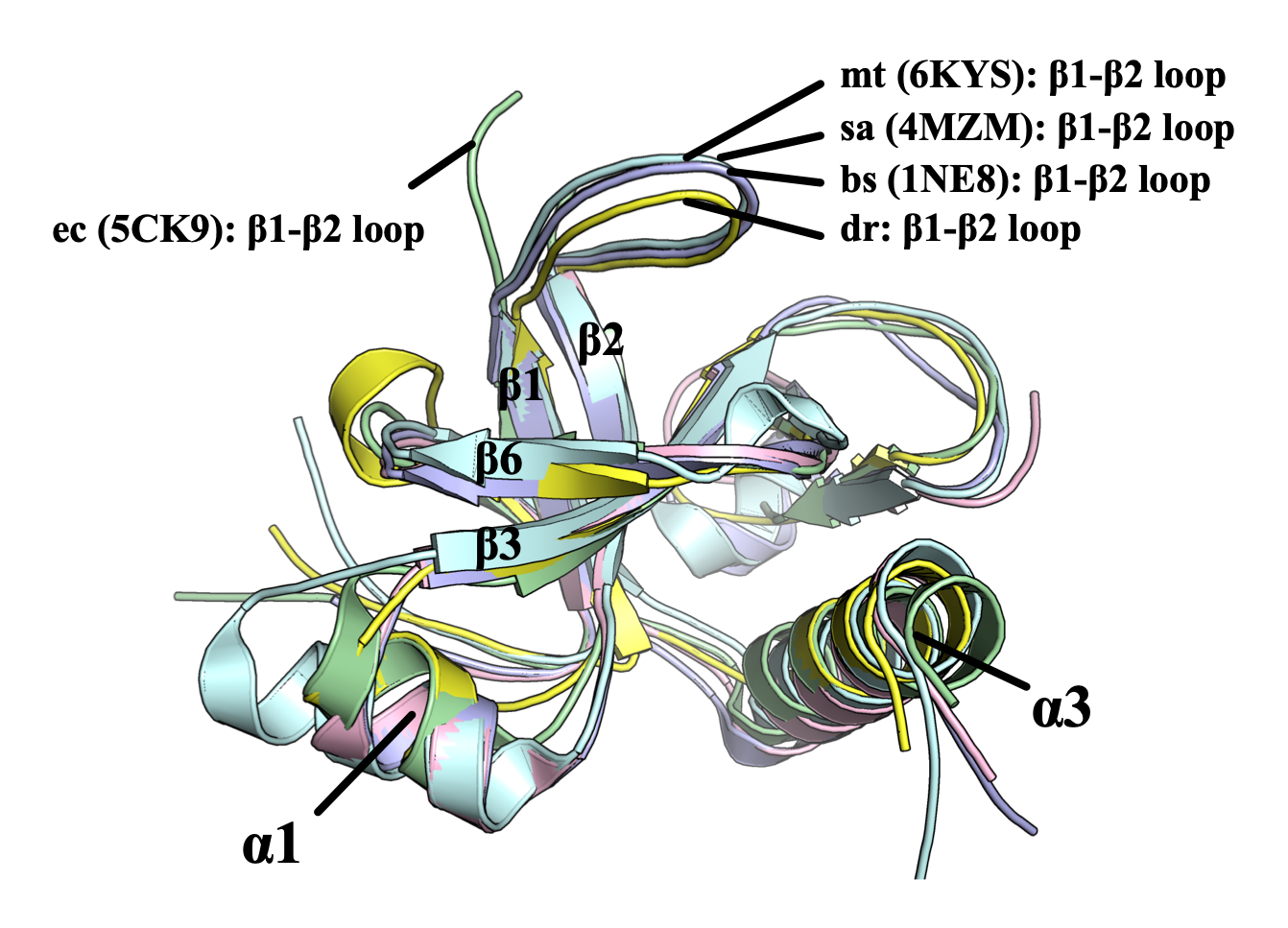


**Figure S3. Expression levels of MazE-dr under 5 and 15 µg/mL MMC treatments.** Equivalent amounts of cell-free extract (10 µg) were loaded in each lane. GroEL was used as a control protein.


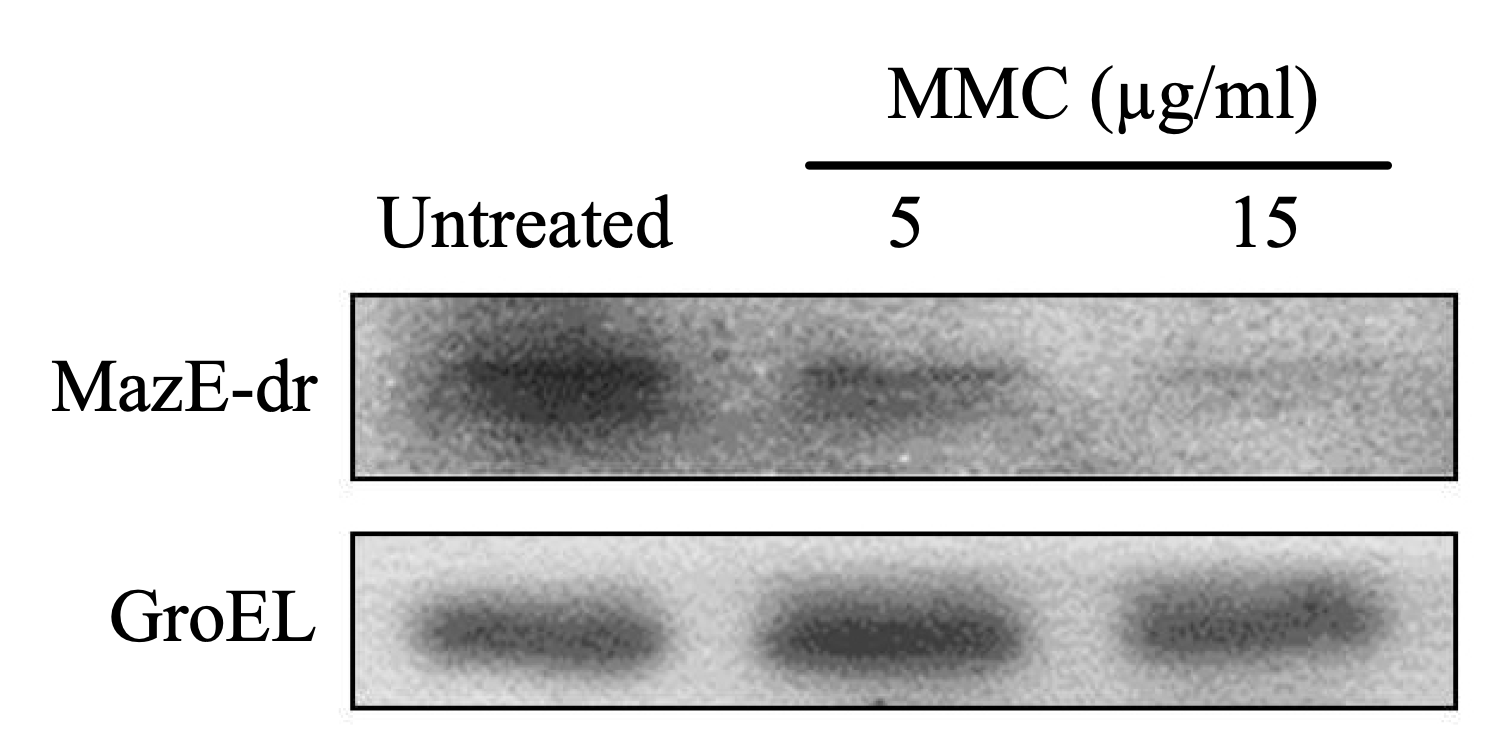

Supplement: Supplementary file 1 [file Data_Sheet_1.docx]
